# Investigating the mechanism of aortic valve stenosis: the role of magnesium salts

**DOI:** 10.1186/1749-8090-8-S1-O18

**Published:** 2013-09-11

**Authors:** V Dritsa, K Pissaridi, J Anastassopoulou, E Koutoulakis, I Mamarelis, Chr Kotoulas

**Affiliations:** 1 Chemical Engineering Department, Radiation Chemistry & Biospectroscopy, National Technical University of Athens, Greece; 2 Department of Cardiology, NIMTS Veteran Hospital of Athens, Greece; 3 Department of Cardiology, 401 Army General Hospital of Athens, Greece; 4 Department of Cardiac Surgery, «Iaso» General Hospital of Athens, Greece

## Background

The calcification ofaortic valves is a common diseaseand valve replacement is the only established treatment. Herewith, we use infrared (FT-IR) spectroscopyto investigate and characterize the mineral deposits in order to understand the mechanism of aortic valve calcification and stenosis.

## Methods

30 aortic valves of patients (65-80 years), who underwent surgical aortic valve replacement due to aortic stenosis, were used. The ATR-FT-IR spectra were recorded with a Nicolet 6700 thermoscientific spectrometer. SEM-EDX and XRD were from Fei Co, the Netherlands and Simens D-500 X-Ray diffractometer, respectively.

## Results

The changes of FT-IR spectraat 1743 cm^-1^resulted from hyperoxidation of lipids due to oxidative stress. The characteristic bands at the spectral regions 1200-900 cm^-1^ and 700-400 cm^-1^showed the formation of low crystallinity biological hydroxyapatite (Ca_10_(PO_4_)_6_(OH)_2_) and calcium monophosphate (CaHPO_4_) salts. The results were confirmed using SEM-ΕDAX and XRD analysis. The observed cross-linking bonds between collagen and elastin were the principal sites for calcium deposition and progression. The findings confirmed the hypothesis that hydroxyapatite is formed predominantly due to ATP cycle, where the release of phosphate anions take place in ischemic pathways.

## Conclusions

The characteristic FT-IR absorption bands of calcified aortic valves showed hyperoxidation of membranes (a pro-inflammation stage), while the mineral deposits were consistent of low crystallinity biological hydroxyapatite, Ca_2_HPO_4_ and calcium phosphates. SEM-EDAX data showed substitution of calcium cations from magnesium cations leading to amorphous salts, preventing thus the aortic valve stenosis. Treatment of these patients with magnesium salts maybe could reduce the progress of aortic valve stenosis after valve replacement.

